# Secondary Syphilis in a 21-Year-Old Woman With Diffuse Mucocutaneous Lesions: A Case Report and Diagnostic Review

**DOI:** 10.7759/cureus.89932

**Published:** 2025-08-12

**Authors:** Wahjuni Sudibyo, Taufiq Qurrohman

**Affiliations:** 1 Dermatology and Venerology, Prof. Dr. Aloei Saboe General Hospital, Gorontalo, IDN; 2 Department of Dermatology, Faculty of Medicine Universitas Negeri Gorontalo, Gorontalo, IDN

**Keywords:** mucocutaneous rash, secondary syphilis, serologic tests, sexually transmitted infection, treponema pallidum

## Abstract

This case report describes a 21-year-old woman who presented with diffuse, non-pruritic erythematous papules involving the palms, soles, cervical region, inguinal folds, and perineal area. The lesions appeared approximately one month following unprotected sexual contact with a partner with multiple previous sexual relationships. Clinical examination revealed well-demarcated, lenticular papules with fine scaling and generalized non-tender lymphadenopathy, without mucosal erosions or genital ulcers. Serologic testing confirmed the diagnosis of secondary syphilis, with high titers on both treponemal and non-treponemal assays. The patient was treated with a single intramuscular injection of benzathine penicillin G and supportive therapy. Early clinical improvement was observed, and follow-up serological monitoring was arranged. This case emphasizes the importance of recognizing the protean manifestations of secondary syphilis, which may mimic other dermatologic or systemic conditions. Prompt diagnosis through comprehensive history-taking, physical examination, and appropriate serological testing is critical. In addition to treatment, counseling and partner notification are essential components of management to prevent reinfection and curb disease transmission. This report highlights the continued relevance of syphilis in modern clinical practice and underscores the importance of maintaining a high index of suspicion, particularly in young, sexually active individuals presenting with unexplained mucocutaneous lesions.

## Introduction

This case report presents a 21-year-old woman who developed diffuse, non-pruritic erythematous papules involving the palms, soles, cervical region, inguinal folds, and perineal area, persisting for approximately three weeks before medical evaluation. The lesions appeared around one month after unprotected sexual contact with a new partner, whose sexual health status was unknown. On clinical examination, the patient exhibited well-demarcated, lenticular papules with fine scaling, along with generalized, non-tender lymphadenopathy. There were no mucosal erosions or genital ulcers. Differential diagnoses considered included pityriasis rosea, viral exanthema, drug eruptions, and psoriasis. Serologic tests, including Venereal Disease Research Laboratory (VDRL) and Treponema pallidum hemagglutination assay (TPHA), confirmed secondary syphilis, with both yielding high titers. The patient received a single intramuscular injection of benzathine penicillin G and supportive therapy, which included antipyretics and skin moisturizers. Clinical improvement was observed within 72 hours, and a one-week follow-up was arranged for serological monitoring. This case underscores the protean manifestations of secondary syphilis, particularly in the absence of mucosal or genital lesions, which may delay diagnosis. Recognizing atypical presentations is essential in the context of the global resurgence of syphilis. Prompt diagnosis through detailed history-taking, physical examination, and targeted serologic testing remains vital. Partner notification and counseling were also undertaken to prevent reinfection and limit transmission.

## Case presentation

A 21-year-old female presented with non-pruritic, symmetric erythematous papules on the palms and soles, which progressively spread over two weeks to involve the cervical region, inguinal folds, popliteal fossae, and perineum. The onset of symptoms occurred approximately four weeks after unprotected sexual intercourse with a former partner, as reported by the patient. She denied any prior systemic illness, recent medication use, drug allergies, constitutional symptoms (e.g., fever, malaise, or weight loss), or family history of sexually transmitted infections or autoimmune conditions.

**Figure 1:**
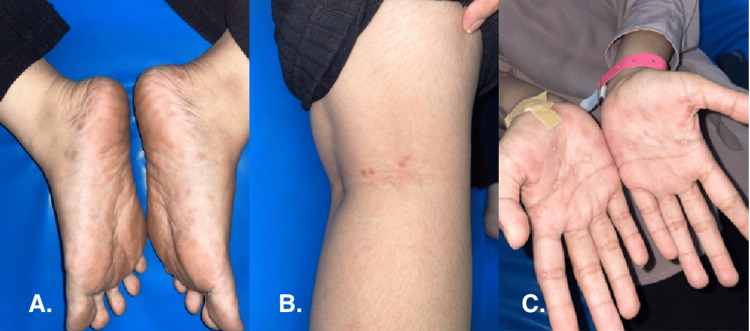
Clinical manifestations when the patient was admitted to the ER, October 7, 2024 Erythematous papules accompanied by lenticular scales in a circumscribed order on (A) the plantar pedis, (B) popliteal fossa, and (C) palmar region.

On examination, the patient was alert, oriented, and hemodynamically stable. Dermatological assessment revealed multiple well-demarcated, lenticular erythematous papules with fine scaling, symmetrically distributed over the palmar surfaces, soles, cervical area, and popliteal fossae, as shown in Figure 1. There was generalized, non-tender lymphadenopathy, most prominent in the cervical and inguinal regions, with nodes measuring approximately 1-1.5 cm, firm in consistency, and mobile. No mucosal erosions, genital ulcers, or oral lesions were noted.

Routine laboratory investigations, including complete blood count and liver function tests, were within normal limits shown in Table 1. Serological testing revealed a reactive VDRL test, a non-treponemal assay, with a titer of 1:16, and a reactive TPHA, a treponemal-specific test, with a titer of 1:5120, as shown in Table 2. HIV screening was performed and returned negative. 

**Table 1:** Hematological Parameters at Presentation All values were within normal reference ranges, with no evidence of anemia, leukocytosis, thrombocytopenia, or macrocytic indices.

Test Name	Result	Reference Range
Hemoglobin (Hb)	12.7 g/dL	12–16 g/dL
Leukocytes	7.8 × 10³/µL	4.0–11.0 × 10³/µL
Platelets	277 × 10³/µL	150–450 × 10³/µL
Hematocrit (PCV)	37%	36–47%
Erythrocytes	4.34 × 10⁶/µL	4.1–5.1 × 10⁶/µL
Mean Corpuscular Volume (MCV)	84 fL	81–99 fL
Mean Corpuscular Hemoglobin (MCH)	29 pg	27–31 pg
Mean Corpuscular Hemoglobin Conc. (MCHC)	35 g/dL	31–37 g/dL
Red Cell Distribution Width – CV (RDW-CV)	12%	11.5–14.5%
Mean Platelet Volume (MPV)	8 fL	6.5–9.5 fL

**Table 2:** Serologic Test Results for Syphilis and HIV The patient demonstrated reactive results on both non-treponemal (VDRL) and treponemal (TPHA) assays, consistent with secondary syphilis. HIV antibody screening was also performed and found to be non-reactive.

Test	Result	Reference Range	Interpretation
VDRL (non-treponemal test)	1:16	Non-reactive	Reactive — active infection
TPHA (treponemal test)	1:5120	Non-reactive	Reactive — confirms infection
HIV Antibody Test	Non-reactive	Non-reactive	Negative for HIV

Based on the characteristic clinical presentation and confirmatory serologic testing, a diagnosis of secondary syphilis was established. Differential diagnoses considered included drug-induced exanthema and pityriasis rosea. However, the symmetric involvement of the palms and soles, the absence of a herald patch or recent drug exposure, and the presence of generalized, non-tender lymphadenopathy favored secondary syphilis. The fine scaling and lenticular morphology of the papules further supported this diagnosis over the alternatives. Additionally, the lack of constitutional symptoms such as fever or malaise made viral exanthem less likely.

The patient was treated according to the Centers for Disease Control and Prevention (CDC) guidelines for secondary syphilis with a single intramuscular injection of 2.4 million units of benzathine penicillin G. Supportive therapy included intravenous fluids and omeprazole 40 mg IV, administered prophylactically to prevent gastric irritation related to fasting and mild nausea during treatment observation. A multivitamin supplement containing B-complex and vitamin C was given based on patient-reported low dietary intake, although no deficiency was formally documented.

Non-pharmacologic management included comprehensive counseling regarding the nature of syphilis, its modes of transmission, and the necessity of notifying and testing sexual partners to prevent reinfection and further spread. At the 48-hour follow-up visit, physician-observed clinical improvement was documented. The erythematous papules showed visible regression, including fading of erythema and early desquamation, particularly over the palms and soles, making it less disturbing for the patient. The patient reported decreased skin sensitivity and resolution of prior discomfort, though she had not experienced pruritus initially. She was scheduled for monthly non-treponemal (VDRL) titer monitoring to evaluate serologic response and treatment efficacy.

## Discussion

Syphilis continues to pose a multifaceted clinical and public health challenge due to its protean manifestations, evolving epidemiology, and sociocultural implications. Secondary syphilis, in particular, exemplifies the complexity of the disease as it represents a hematogenous dissemination phase, where Treponema pallidum spreads via the bloodstream, resulting in systemic involvement. This phase often occurs four to 10 weeks after the initial chancre of primary syphilis heals and is considered the most immunologically active and symptomatic stage of the infection [1]. In the case presented, the polymorphous rash with prominent acral involvement, including the palms and soles, was a key diagnostic feature. Such rashes can be erythematous, maculopapular, or even psoriasiform, and they often mimic more common dermatologic conditions such as pityriasis rosea, psoriasis, lichen planus, seborrheic dermatitis, or tinea corporis [2]. The symmetrical distribution of lesions, particularly on intertriginous areas like the cervical and inguinal folds, is a classic but frequently overlooked clue to secondary syphilis. Secondary syphilis reflects an intense immune response, particularly driven by T-helper 1 (Th1) cells and macrophages, which respond to the systemic spread of the spirochetes [3,4]. These immune mechanisms contribute to the wide array of mucocutaneous findings and systemic symptoms such as malaise, lymphadenopathy, low-grade fever, myalgia, and arthralgia. In more severe cases, syphilis can cause hepatitis (syphilitic hepatitis), glomerulonephritis, iritis, and neurological symptoms even in the early stages. In the patient described, the absence of oral or genital lesions initially obscured suspicion for a sexually transmitted etiology. This underscores the importance of maintaining a high index of suspicion, especially in sexually active individuals presenting with unexplained dermatological findings. Laboratory confirmation through serologic testing remains the cornerstone of diagnosis. Non-treponemal tests like the VDRL test are useful for both screening and monitoring treatment response, while treponemal tests such as Treponema pallidum particle agglutination (TPPA) and fluorescent treponemal antibody absorption (FTA-ABS) confirm the diagnosis [3-6]. Treatment with a single dose of intramuscular benzathine penicillin G (2.4 million units) is highly effective for early syphilis. The success of therapy is monitored through follow-up VDRL titers, with a fourfold decline in titers at six months considered an adequate response [7,8]. In penicillin-allergic individuals, doxycycline or azithromycin may be considered, although resistance is an emerging concern. Importantly, treatment should be accompanied by sexual health counseling, including partner notification and screening [7]. Beyond clinical management, the psychosocial context must not be neglected. Syphilis remains highly stigmatized, particularly among adolescents and young adults. Fear of judgment, lack of anonymity, and misinformation can deter individuals from seeking care [9]. In our case, the patient initially delayed consultation, attributing the rash to a common skin condition. This highlights the need for culturally sensitive, destigmatized communication in clinical and community settings. School-based and online sexual health education could be particularly impactful in such demographic groups. Finally, it is imperative to frame syphilis within the broader public health context. The global resurgence of syphilis, including among women of reproductive age, poses risks for congenital transmission. Public health guidelines from the WHO recommend routine antenatal screening for syphilis to prevent adverse pregnancy outcomes. Furthermore, syndromic management alone may be insufficient, especially in cases with atypical presentations, thus requiring laboratory-based surveillance and capacity building for health workers [10].

## Conclusions

In conclusion, this case underscores the diagnostic challenges posed by secondary syphilis, particularly due to its protean mucocutaneous manifestations and clinical overlap with other dermatologic conditions. Accurate diagnosis requires an integrated clinical and diagnostic approach, encompassing thorough history-taking, targeted physical examination, and confirmatory serological testing. Furthermore, comprehensive management should include patient education, partner notification and treatment, psychosocial support, and structured follow-up to ensure therapeutic success. Strengthening these components not only improves individual patient outcomes but also plays a critical role in reducing syphilis transmission and advancing broader public health efforts toward infection control.

## References

[1] Lafond RE, Lukehart SA (2006). Biological basis for syphilis. Clin Microbiol Rev.

[2] Workowski KA, Bachmann LH, Chan PA (2021). Sexually transmitted infections treatment guidelines, 2021. MMWR Recomm Rep.

[3] Mindel A, Tovey SJ, Timmins DJ, Williams P (1989). Primary and secondary syphilis, 20 years' experience. 2. Clinical features. Genitourin Med.

[4] Seña AC, White BL, Sparling PF (2010). Novel Treponema pallidum serologic tests: a paradigm shift in syphilis screening for the 21st century. Clin Infect Dis.

[5] Clement ME, Okeke NL, Hicks CB (2014). Treatment of syphilis: a systematic review. JAMA.

[6] (2024). WHO guidelines for the treatment of Treponema pallidum (syphilis). https://www.who.int/publications/i/item/who-guidelines-for-the-treatment-of-treponema-pallidum-(syphilis).

[7] Tucker JD, Bien CH, Peeling RW (2013). Point-of-care testing for sexually transmitted infections: recent advances and implications for disease control. Curr Opin Infect Dis.

[8] Heymans R, van der Helm JJ, de Vries HJ (2012). The role of molecular typing of Treponema pallidum in syphilis control. Clin Microbiol Infect.

[9] Zetola NM, Klausner JD (2007). Syphilis and HIV infection: an update. Clin Infect Dis.

[10] Stamm LV (2016). Syphilis: re-emergence of an old foe. Microb Cell.

